# A congenital cystic pulmonary airway malformation occurring together with both an extralobar pulmonary sequestration and an esophageal duplication cyst

**DOI:** 10.1002/ccr3.2455

**Published:** 2019-09-30

**Authors:** Lindel C. Dewberry, Andrew Trecartin, Csaba Galambos, Sarah A. Hilton, Kimberly Dannull, Michael V. Zaretsky, Nicholas Behrendt, Henry L. Galan, Ahmed I. Marwan, Kenneth W. Liechty

**Affiliations:** ^1^ Department of Surgery Laboratory for Fetal and Regenerative Biology University of Colorado Denver School of Medicine and Children's Hospital Colorado Aurora Colorado; ^2^ Division of Pediatric Surgery Department of Surgery Children's Hospital of Colorado Aurora Colorado; ^3^ Department of Pathology University of Colorado School of Medicine and Children's Hospital Colorado Aurora Colorado; ^4^ Department of Radiology University of Colorado Denver School of Medicine and Children's Hospital Colorado Aurora Colorado; ^5^ Colorado Fetal Care Center Colorado Institute for Fetal & Maternal Health Children's Hospital of Colorado Aurora Colorado; ^6^ Division of Maternal Fetal Medicine University of Colorado Denver School of Medicine and Children's Hospital Colorado Aurora Colorado

**Keywords:** cystic lung disease, cystic pulmonary airway malformation, esophageal duplication cyst, extralobar pulmonary sequestration, prenatal imaging

## Abstract

A foregut duplication cyst occurring together with both a congenital cystic pulmonary airway malformation and extralobar pulmonary sequestration is an unusual combination. Prenatal ultrasound, MRI, and postnatal CT are helpful for operative planning. Surgical resection is the definitive management for all three anomalies.

## Graphical abstract

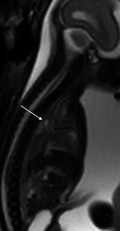

## 1 INTRODUCTION

This report describes a case of an infant with prenatally diagnosed complex congenital lung lesion. At the time of surgery, three distinct lesions were discovered, including extralobar pulmonary sequestration. Pathology demonstrated a congenital cystic pulmonary airway malformation occurring together with both an extralobar pulmonary sequestration and foregut duplication cyst.

Cystic lung disease (CLD) occurs in 1/10 000 to 1/35 000 births, and the incidence continues to rise due to advances in prenatal imaging^1^ with improved prenatal detection. Two concurrent lesions are rare, especially involving a noncommunicating esophageal duplication cyst. This should be differentiated from bronchopulmonary foregut malformations where there is communication between the lung and foregut lesions.^2^

## 2 CASE

A 31‐year‐old woman G1P0 with no significant past medical history was referred to institution's fetal center due to a chest lesion on ultrasound. The prenatal ultrasound at 32‐week gestational age demonstrated an echogenic lesion in the right thorax with the cluster of several small cysts and an area of overinflation believed to represent a congenital pulmonary airway malformation (CPAM) with mixed microcystic‐macrocystic features. The CPAM volume ratio (CVR) was 0.6 (CVR greater than 1.6 has been shown to be correlated with hydrops). An ellipse volume formula is used and divided by the head circumference to correct for differences of gestational age.^3^

**Figure 1:**
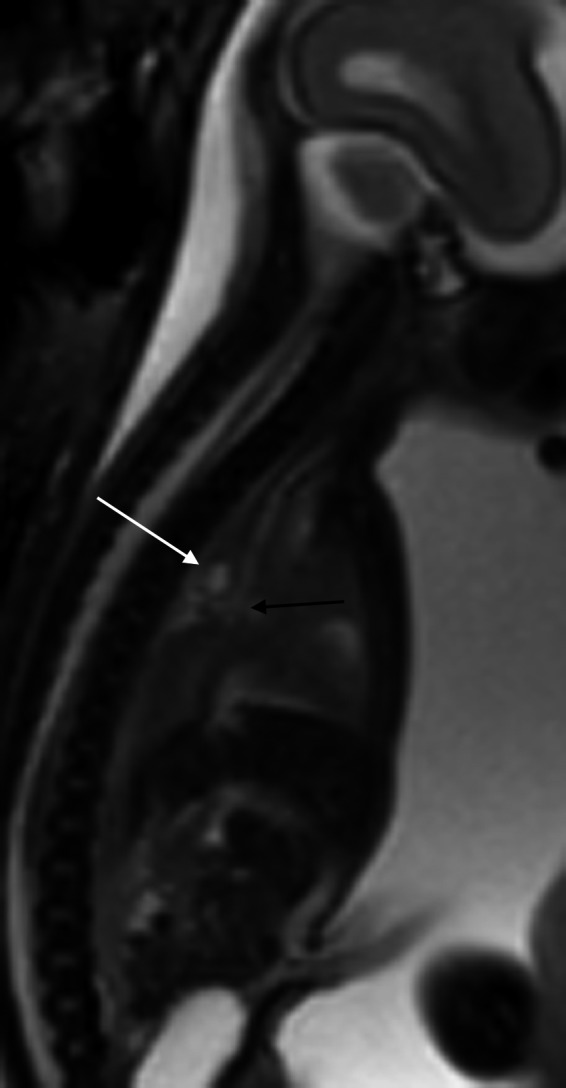
Midline sagittal SS‐FSE MRI sequence of the fetal chest and abdomen demonstrating the relationship between the carina (black arrow) and the foregut duplication cyst (white arrow)

**Figure 2:**
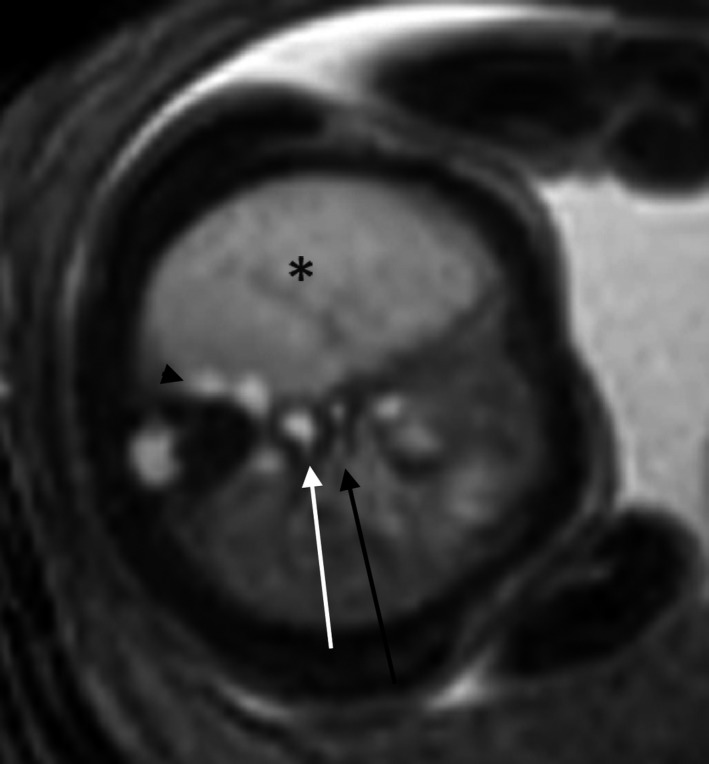
Axial SSFP MRI sequence of the fetal chest demonstrating the relationship of the carina (black arrow), foregut duplication cyst (white arrow), partially imaged cluster of small cysts which extended further inferior (black arrowhead), and the area of right midlung overinflation (asterisk)

**Figure 3:**
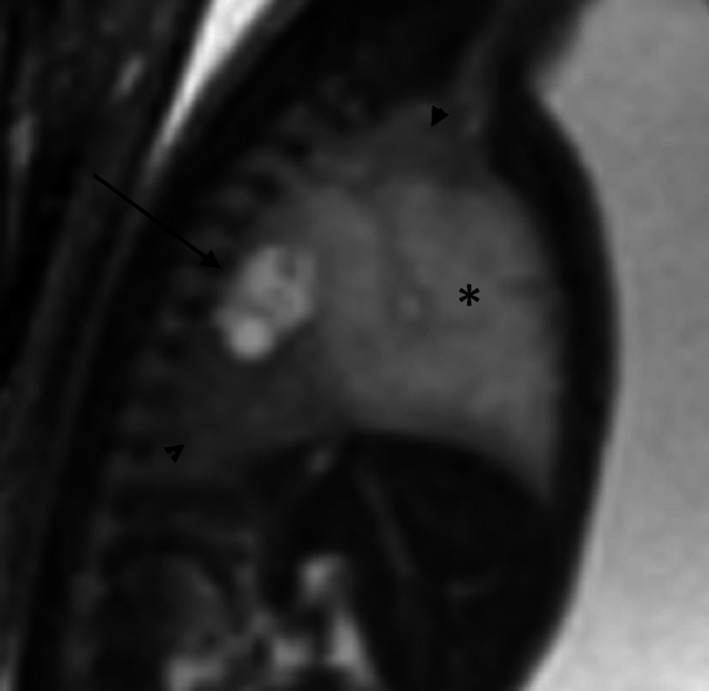
Right parasagittal SSFP MRI sequence of the fetal chest demonstrating the relationship of the cluster of small cysts (black arrow) with the mildly compressed normal lung (black arrow heads) and the region of T2 hyperintensity and overinflation (asterisk)

Fetal magnetic resonance imaging (MRI) at 25 gestational weeks demonstrated a mixed right lung lesion with a 4.5 mm cyst located between the carina and the esophagus, suggesting a foregut duplication cyst (Figure 1). There was a cluster of small lung cysts posteriorly and inferiorly, the largest measuring 6 mm, most likely representing a CPAM; within the right midlung, there was homogeneous T2 hyperintensity and overinflation (Figures 2 and 3). No systemic vessel was identified to suggest sequestration. A fetal echocardiogram was normal.

The patient was born at term and was asymptomatic after birth. A routine computed tomography angiogram (CTA) scan was performed at 3 months. This examination demonstrated the foregut duplication cyst posterior to the carina and the small cluster of cysts further posterior and inferior. There was wedge‐shaped area of pulmonary hyperinflation in the right midlung in the region of prenatal fluid trapping, unchanged in size from prenatal imaging relative to the size of the chest (not shown). No systemic vessel was identified.

**Figure 4:**
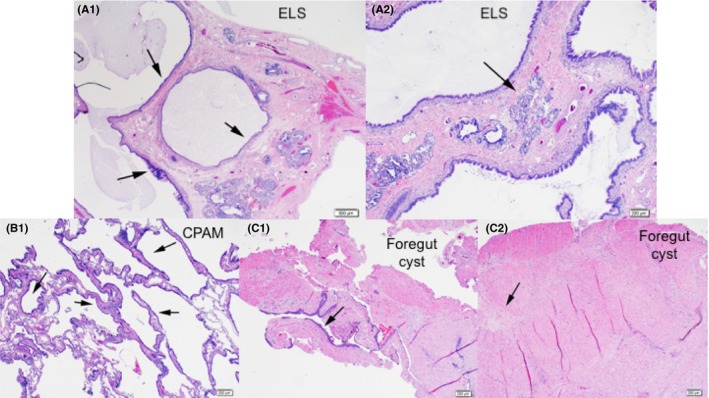
A‐C, Specimen A (ELS). Low power micrograph (A1, H + E stain, 2× magnification) demonstrates large cysts (largest about 1.5 cm, arrows) are present throughout lined by ciliated columnar epithelium with intervening broad fibrous septa. Mucous material is present within some cyst. The cyst wall contains smooth muscle and cartilage. At medium power (A2, HE, 4×) minimal alveolar development between the cysts is noted (arrow). There is a tail‐like soft tissue attached to the pleura of one fragment that contains thick‐walled muscular arteries consistent with systemic arteries (feeding vessels, not shown). These features are diagnostic of extralobar sequestration. Specimen B (CPAM). Medium power micrograph (B1, H + E, 4× magnification) shows maldeveloped lung parenchyma with smaller cysts (up 0.5 cm) focally distributed in a near back to back fashion and lined by ciliated respiratory epithelium (arrows). Smooth muscle but no cartilage is present within the cyst wall. Between cysts there are enlarged and simplified alveoli. These features are most consistent with CPAM type 2 lesion. Specimen C (Foregut Cyst). Medium power micrographs demonstrate abundant fibromuscular tissue partially line by a combination of squamous and respiratory epithelium (C1, arrow). An outline of myenteric plexus is noted within layers of smooth muscle bundles (C2, arrow). These features are most consistent with a foregut duplication cysts

The patient was taken to the operating room at 3 months of age and underwent a muscle‐sparing thoracotomy. Upon inspection, the right lung was normally trilobed. There was an additional collapsed extralobar sequestration with a small feeding vessel from an intercostal artery which was resected. The foregut duplication cyst was demonstrated to be within the thoracic esophagus. The patient then underwent a right upper lobe resection for the CPAM, and the esophageal duplication cyst was enucleated without violation of the underlying mucosa. A 12fr chest tube was left in place. The chest tube remained in place for 4 days, and the patient was discharged home postoperative day 5 after an uncomplicated hospital course. The patient has continued to do well on outpatient follow‐up. Final pathology demonstrated a complex mixed lung malformation, including an extralobar sequestration, CPAM type 2, and intraesophageal duplication cyst of mixed bronchogenic and esophageal type (Figure 4A‐C).

## 3 DISCUSSION

This case highlights the diagnosis and management of three concurrent congenital anomalies: foregut duplication cyst, CPAM, and extralobar pulmonary sequestration. As these three malformations did not communicate with one another, they were not classified as a true bronchopulmonary foregut malformation (BPFM), but likely have embryological similarities. BPFMs arise as supranumery buds from the primitive foregut, and the eventual type of BPFM is related to the embryological stage in which it arose, the direction of aberrant tissue growth, and the relationship to the parent tissue, structure, and viscous.^4^, ^5^, ^6^, ^7^ Foregut duplication cysts encompass a spectrum of pathology with a mucosal lining of respiratory tract, alimentary tract, or both, and they may or may not be attached or adjacent to the esophagus or bronchus.^8^

We have previously described an association between an extrapulmonary lobar sequestration and a bronchogenic cyst.^9^ Other studies have also described complex bronchopulmonary foregut malformations of the mixed bronchogenic and esophageal type with presentation ranging from asymptomatic infant to adults with recurrent infections.^6^, ^10^, ^11^, ^12^, ^13^, ^14^

A BPFM consisting of an extralobar pulmonary sequestration containing both ciliated columnar epithelium adjacent to cartilage with stratified squamous epithelium over smooth muscle was found to be connected to the esophagus by a stalk in one report.^15^ In our case, the extralobar pulmonary sequestration was connected to the mediastinum by a stalk adjacent to the esophageal duplication cyst, but not clearly connected to it. The CPAM in our case was within the right upper lobe of the lung and had no anatomic association with the sequestration or the esophageal duplication cyst.

A discrete foregut duplication cyst, CPAM, and extralobar pulmonary sequestration occurring in the same patient are rarely encountered in the literature. A single mass containing a esophageal duplication cyst, CPAM, extralobar sequestration, and benign teratoma has been reported.^16^ This was identified on final pathology of the excised specimen. However, our patient did not have a single mass, but rather demonstrated each of the separate three entities—foregut duplication cyst, CPAM, and extralobar sequestration—with no anatomic connection to each other. An association between bronchogenic and esophageal duplication cysts has been established,^17^ and however, a CPAM occurring together with an esophageal duplication cyst is quite rare.^18^ An esophageal duplication cyst lined by respiratory epithelium in association with a CPAM containing metaplastic changes in a 6‐year‐old was reported.^18^ Others have reported esophageal duplication cysts lined by squamous esophageal epithelium occurring with CPAMs lined by pseudostratified columnar epithelium.^19^, ^20^ The foregut duplication cyst in our case contained both respiratory and esophageal epithelium as has been previously described.^11^, ^21^ Esophageal and foregut duplication cysts are histologically similar and are differentiated intraoperatively. It is possible that mechanical factors such as bronchial obstruction by the duplication cyst in utero played a role in the development of the CPAM.

Clinical presentation of cystic lung disease varies depending on the size and location of the malformation. Mass effect may lead to pain or discomfort related to size, dysphagia from esophageal compression, or respiratory symptoms due to airway obstruction. Furthermore, if the lining of a foregut malformation is comprised of gastric mucosa, this may lead to perforation and bleeding.^8^ Rarely, these cases present as fetal hydrops due to vascular compression. However, with the advent of advanced fetal imaging, most of these lesions are diagnosed prenatally and patients are asymptomatic postnatally. Prenatal ultrasonography serves as a useful diagnostic modality with a high sensitivity of 81%‐93% but poor specificity of 32% for CCAM detection.^22^, ^23^ Postnatally, a CT scan is considered standard of care to confirm the continued presence of the mass, further define its anatomy and determine whether there is extension into the abdomen. A contrast study may be considered to evaluate for communication between a duplication cyst and the esophagus, although this is rare.

Surgery is recommended for all complex foregut malformations due to the risk of malignancy^24^ as well as increased difficulty and risk with resection due to inflammation if surgery is delayed until the patient becomes symptomatic with recurrent infections.^25^ For CPAMs specifically, surgical resection is recommended, but the timing remains controversial. Timing of surgical resection is dependent on symptoms. Symptomatic patients generally undergo surgery earlier. Other arguments for earlier surgical resection include the benefits of compensatory lung growth at a younger age as well as avoiding the radiation of follow‐up scans to monitor the lesion. The classic surgical approach has been through open thoracotomy^26^ although equivalent results with thoracoscopic surgery have been published.^27^

## 4 CONCLUSION

A foregut duplication cyst occurring together with both a congenital cystic pulmonary airway malformation and extralobar pulmonary sequestration is an unusual combination. Prenatal ultrasound, MRI, and postnatal CT are helpful for operative planning, but sometimes cannot provide complete diagnostic accuracy. A multidisciplinary approach aids in management, and surgical resection is the definitive management for all three anomalies.

## CONFLICT OF INTEREST

None declared.

## AUTHOR CONTRIBUTIONS

LD: made substantial contributions to the conception and design, acquisition of data, interpretation of data, been involved in the drafting of the manuscript, and revised it critically for important intellectual content. AT: made substantial contributions to the conception and design, interpretation of data, been involved in the drafting of the manuscript, and revised it critically for important intellectual content. CG: made substantial contributions to the conception and design, acquisition of data, and revised it critically for important intellectual content. SH: made substantial contributions to interpretation of data and revised it critically for important intellectual content. KD: made substantial contributions to the conception and design, acquisition of data, and revised it critically for important intellectual content. MZ: made substantial contributions to interpretation of data and revised it critically for important intellectual content. NB: made substantial contributions to interpretation of data and revised it critically for important intellectual content. HG: made substantial contributions to interpretation of data and revised it critically for important intellectual content. AM: made substantial contributions to interpretation of data and revised it critically for important intellectual content. KL: made substantial contributions to the conception and design, acquisition of data, and revised it critically for important intellectual content. All authors have given final approval of the version to be published and agreed to be accountable for all aspects of the work in ensuring that questions related to the accuracy or integrity of any part of the work are appropriately investigated and resolved.
